# Real-World Perspectives From Surgeons and Oncologists on Resectability Definition and Multidisciplinary Team Discussion of Stage III NSCLC in People’s Republic of China, Hong Kong, and Macau: A Physician Survey

**DOI:** 10.1016/j.jtocrr.2022.100308

**Published:** 2022-03-19

**Authors:** Victor Ho-Fun Lee, Joseph Siu Kie Au, Ju-Wei Mu, Guangli Xiao, Fiona Mei Ying Lim, Hon Chi Suen, Kam Hung Wong

**Affiliations:** a Department of Clinical Oncology, Li Ka Shing Faculty of Medicine, The University of Hong Kong, Hong Kong, People’s Republic of China; b Oncology Centre, Hong Kong Adventist Hospital, Hong Kong, People’s Republic of China; c Department of Thoracic Surgery, Cancer Hospital, Chinese Academy of Medical Sciences, Beijing, People’s Republic of China; d Radiation Therapy Center, Kiang Wu Hospital, Macao, People’s Republic of China; e Department of Oncology, Princess Margaret Hospital, Hong Kong, People’s Republic of China; f Hong Kong Cardiothoracic Surgery, Hong Kong, People’s Republic of China; g Department of Clinical Oncology, Queen Elizabeth Hospital, Hong Kong, People’s Republic of China

**Keywords:** Non–small cell lung cancer, Stage III NSCLC, Surgery, Chemotherapy, Radiotherapy, Multidisciplinary management

## Abstract

**Introduction:**

Decision-making in diagnosis and management of stage III NSCLC remains complex owing to disease heterogeneity and diverse treatment options, and often warrants multidisciplinary team discussion. Specifically, the selection of patients for multimodality approaches involving surgical resection presents notable challenges owing to heterogeneity in guideline definitions and the subjective, case-specific nature of evaluating resectability on the basis of preoperative assessments.

**Methods:**

An internet- and paper-based survey was conducted in 2020 among lung cancer specialists in the People’s Republic of China, Hong Kong, and Macau. This survey captured perspectives on stage III NSCLC on real-world diagnosis/staging practice, definition and evaluation of resectability using case scenarios, and preferred treatment paradigms.

**Results:**

A total of 60 completed responses were obtained (60.0% surgeons; 40.0% oncologists). The surgeons’ and oncologists’ responses differed most in the assessment of resectability in specific case scenarios despite overall agreement on top factors determining resectability (T stage, lymph node size, and lymph node location). Of the 17 scenarios, specialists agreed (≥80%) on four “resectable” and six “unresectable” scenarios; of the seven scenarios with less than 80% agreement, surgeons and oncologists had diverging responses for six scenarios. Multidisciplinary team discussions were available in most of the respondents’ institutions but usually covered only selected (<50%) stage III cases.

**Conclusions:**

This survey used a comprehensive set of stage III NSCLC case scenarios to understand how working definitions of resectability may differ between surgeons and oncologists, and thus, identify types of cases to prioritize for multidisciplinary discussions to maximize limited resources. In parallel, the development of a multidisciplinary expert consensus on treatment approaches could complement local institutional expertise as a reference for decision-making.

## Introduction

Stage III or locally advanced NSCLC accounts for around 30% of NSCLC cases worldwide.^1^ The eighth edition^2^ of the American Joint Committee on Cancer/Union for International Cancer Control TNM staging system for lung cancer classifies stage III NSCLC into stages IIIA, IIIB, and IIIC, considered relatively distinct in terms of their prognosis and long-term outcomes.^1^^,^^2^ Yet, within these subgroups, there is clinically important variability in disease burden and presentation, which can determine the choice of multimodality treatment combining locoregional (surgery, radiotherapy) and systemic therapy. For example, surgical resection may or may not be considered an option for patients with stage IIIA NSCLC, depending on the specific features of each case.^3^^,^^4^ Appropriate selection of patients for multimodality approaches involving surgical resection presents notable challenges, partly owing to heterogeneity in guideline definitions and to the subjective and case-specific nature of determining potential resectability on the basis of preoperative assessments.^4^, ^5^, ^6^

Moreover, with the rapidly evolving treatment landscape and emerging treatment options, the management of stage III NSCLC may become even more complex. Readouts from recent/ongoing trials incorporating targeted therapy or immunotherapy into neoadjuvant or adjuvant treatment combined with surgery suggest that some of these may represent future options for patients with operable NSCLC and actionable mutations.^7^, ^8^, ^9^, ^10^ For example, the ADAURA trial revealed significant disease-free survival benefit with adjuvant osimertinib versus placebo in patients with resected EGFR-mutated NSCLC,^7^ leading to the U.S. Food and Drug Administration approval for this indication; osimertinib with or without chemotherapy is also being studied in the neoadjuvant setting (NeoADAURA; NCT04351555). Conversely, other studies highlight the need for careful patient selection for other treatment modalities: the LungART and PORT-C studies both indicate that radiotherapy after complete resection in stage IIIA-N2 does not improve disease-free survival.^11^^,^^12^

Currently, treatment paradigms are more standardized for NSCLC that is deemed unresectable. For unresectable stage III NSCLC, assuming good performance status (PS), concurrent chemoradiotherapy (cCRT) followed by consolidation immunotherapy is the current standard of care.^13^^,^^14^ The PACIFIC trial revealed that immunotherapy with durvalumab improved progression-free survival and overall survival (OS) when used as the consolidation regimen after cCRT,^15^^,^^16^ and European Society for Medical Oncology and National Comprehensive Cancer Network guidelines now recommend immunotherapy for consolidation in unresectable stage III NSCLC, assuming there is sufficient PS.^13^^,^^14^

In contrast, for potentially resectable stage III NSCLC, no universally accepted standard of care exists. Within a multimodality treatment plan, the choice of surgery as locoregional therapy depends on findings from preoperative assessment and the estimated probability of achieving complete resection in each case. Other patient- and treatment-related factors such as PS, comorbidities, technical and functional resectability, and local expertise are also considered relevant in determining resectability.^14^^,^^17^, ^18^, ^19^, ^20^ Among clinicians, the weight given to such factors in real-life decision-making may vary according to specialty, training, practice setting, or access to treatment modalities.

Determining potential resectability is central to appropriate treatment selection for stage III NSCLC, but defining resectability objectively remains challenging, with varied definitions across guidelines.^4^^,^^5^^,^^20^ Besides the T stage, lymph node (LN) extent/location and LN volume/appearance are often discussed in guidelines as considerations for determining whether surgery may be appropriate (reviewed elsewhere^4^^,^^5^). N2 disease, defined as ipsilateral with or without subcarinal mediastinal LN involvement,^2^ encompasses a range of presentations, including incidental/occult N2, single- or multizone, single- or multilevel, bulky or nonbulky LN involvement, some of which have been linked to prognosis and outcomes.^21^^,^^22^ Accurate description of mediastinal LN involvement is, thus, considered crucial for evaluating resectability^20^^,^^23^^,^^24^ and selecting treatment. However, some ambiguity can arise when interpreting guideline recommendations owing to incomplete alignment among descriptive systems using zones versus nodal stations.^4^^,^^5^ In addition, although guidelines concur that bulky N2 disease generally indicates unresectable disease,^14^^,^^17^^,^^20^^,^^25^, ^26^, ^27^ only a few define this in terms of specific nodal volume or dimensions (greatest short-axis diameter ranging from >2.5 cm^20^ to >3 cm^14^); others do not specify a size cutoff but mention other morphologic characteristics of LNs.^26^ In the context of diagnostic workup and staging, it is, therefore, important to perform thorough radiological staging and pathologic LN staging using improved ultrasound-guided methods such as endobronchial ultrasound (EBUS)–guided transbronchial needle aspiration (TBNA) and endoscopic ultrasound-guided fine-needle aspiration.^14^^,^^17^^,^^19^^,^^24^ However, even with thorough investigations, preoperative evaluation of resectability remains subjective because it involves clinical judgment on whether clear resection margins can be achieved in each case.

In the lack of objective and universally accepted criteria for resectability, a thorough evaluation by a multidisciplinary panel or tumor board assumes greater importance and is recommended by guidelines and expert consensus groups.^14^^,^^17^^,^^19^ Such panels should ideally include a range of specialists, including thoracic surgeons, pulmonologists, pathologists, radiologists, medical oncologists, radiation oncologists, and palliative care specialists.^28^ Given the complexities of diagnosis, staging, and management of stage III NSCLC, multidisciplinary team (MDT) discussion was proven to significantly increase median survival in this setting by 15.5 months,^29^ but the current implementation of MDT discussions in reviewing stage III NSCLC cases in the People’s Republic of China has not been specifically described. Multidisciplinary management of NSCLC involves a similar range of specialties (e.g., surgeons, radiation oncologists, medical oncologists, and radiologists) in the People’s Republic of China as mentioned elsewhere.^30^ Public hospitals account for the most inpatient care, although the role of private hospitals has increased over the past decade.^31^ Some analyses indicate that the organization of medical care functions is broadly similar in public and private hospitals in the People’s Republic of China, but that private hospitals are generally smaller and less well-resourced than public hospitals.^31^^,^^32^ To explore potential variation across practice settings and specialties, we surveyed lung cancer specialists practicing in the People’s Republic of China, Hong Kong, and Macau. We sought to understand real-world practice and diversity of clinical opinions on disease staging, assessment of resectability, multimodality treatment approaches, and access to and participation in MDT discussions. Specifically, we presented a comprehensive set of case scenarios to invite respondents to define resectability. The survey results can inform on assessment of resectability and its key considerations and staging and treatment approach in this region. This would support the appropriate selection of patients for multimodality treatment and identify key areas to be targeted in the future to develop an expert consensus on NSCLC management.

## Materials and Methods

### Survey Design and Respondents

This was a self-administered, cross-sectional, internet-based, or paper-based survey conducted from May to August 2020. The internet-based survey was distributed to a list of respondents generated from institutional physician databases and targeted thoracic surgeons, medical oncologists, radiation oncologists, clinical oncologists, and respiratory physicians/pulmonologists involved in treating patients with lung cancer in the People’s Republic of China, Hong Kong, and Macau. The target number of complete responses was 40 (minimum) to permit adequate descriptive summary statistics for the categorical response data to be generated. Ethics approval was granted by the institutional review board of the University of Hong Kong/Hospital Authority Hong Kong West Cluster before survey commencement (UW19-669). Participation was voluntary. Part I of the survey included an introduction to the study and informed consent, followed by part II (questionnaire). The research was conducted according to the principles of the Declaration of Helsinki and locally applicable requirements.

### Questionnaire

This self-report survey was designed to capture clinical opinion on staging practice, resectability, and prevailing treatment paradigms for stage III NSCLC in respondents’ clinical practice. The questionnaire consisted of 37 multiple-choice questions and 17 case scenarios, each with four identical scenario-based questions, inviting respondents to define resectability (Supplementary Table 1). Selected questions had an open-ended option for respondents to provide additional answers. The questionnaire was estimated to take around 20 minutes to complete.

Before implementation, two practicing physicians (VHFL and JSKA) assessed the face validity of the English-language questionnaire and flagged items that were not readily understood or could potentially be misinterpreted. The questionnaire was then translated into simplified Chinese, with back-translation performed to ensure consistency with the English version.

The survey was administered primarily as an internet-based survey, with a paper-based version for distribution to respondents at conferences. The internet-based survey (https://www.doctorcare.hk/hkpos2020/en) was managed using a custom-developed, secure online platform for data collection and storage. Each potential respondent received an e-mail link with instructions and a password to allow access to the survey. Respondents were not required to provide personally identifying information when completing the survey. Several measures were taken to minimize bias in the survey including efforts to encourage diversity in the specialty of the physicians invited to participate.

### Statistical Analysis

Responses were summarized descriptively using frequencies and percentages (categorical variables), overall and by-respondent characteristics. Associations between survey responses and respondent characteristics (clinical specialty: surgeons/oncologists; institution type: government/university teaching hospital, or private hospital/clinic) were evaluated using the chi-square test or Fisher’s exact test, as appropriate. Statistical analyses were performed using Statistical Package for the Social Sciences version 24 (IBM Corp., Armonk, New York). A significance level of 0.05 (2-tailed test) was used.

## Results

A total of 60 completed responses were provided by respondents from South China (63.3%), Macau (25.0%), Hong Kong (8.3%), and North China (3.3%). A total of 36 respondents (60.0%) were thoracic surgeons and the remaining 24 (40.0%) were oncologists (clinical, medical, or radiation oncologists). Most respondents (66.7%) had more than 20 years of experience in their specialty, 66.7% worked in public institutions (government or university teaching hospitals) and 33.3% of respondents worked in private institutions (private hospitals/clinics).

Among thoracic surgeons, 50.0% reported stage IIIA cases were most common in their practice, whereas 79.2% of oncologists reported stage IIIB NSCLC was most often presented (*p* < 0.001) (Supplementary Fig. 1*A*). Over half of surgeons (58.3%) estimated that 10% to 30% of their cases were resectable, whereas 75.0% of oncologists estimated less than 10% of their cases were resectable (*p* < 0.001) (Supplementary Fig. 1*B*). A total of 86% of surgeons and 100% of oncologists reported that 30% or more of stage III cases in their practice were staged as N2 (Supplementary Fig. 1*C*). Most surgeons (66.7%) reported 10% to 50% of their N2 cases received surgery; in contrast, 79.2% of oncologists reported less than 10% of their N2 cases received surgery (*p* < 0.001) (Supplementary Fig. 1*D*). Compared with surgeons, a significantly higher proportion of oncologists routinely performed bronchoscopy and TBNA for every patient or selected patients (87.5% versus 69.4%, *p* = 0.002). In contrast, significantly higher proportions of surgeons versus oncologists routinely performed brain computed tomography (CT) for every patient or selected patients (61.1% versus 20.9%, *p* = 0.007) and positron emission tomography-CT (PET-CT) (72.2% versus 33.3%, *p* = 0.006). Mediastinoscopy was not routinely performed (Supplementary Fig. 2). Over 65% of respondents would test individual biomarkers during initial diagnostic workup for stage III NSCLC (EGFR, *ALK, ROS1, KRAS*, programmed death-ligand 1) (Supplementary Table 2). Gene panels (5–10 genes) and next-generation sequencing were selected by less than 40% of respondents.

### Relative Importance of Factors Determining Resectability

**Figure 1:**
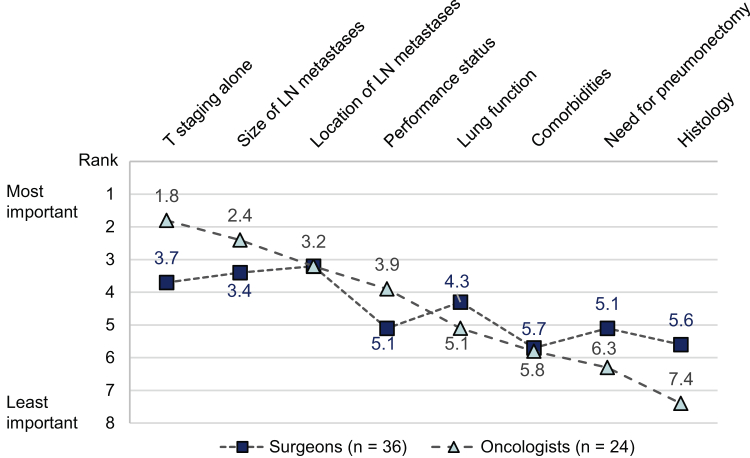
The relative importance of factors determining resectability. The ranking was on the basis of the response to the question “Rate the following factors in determining resectability in stage III setting”: rank 1 being most important, rank 8 being least important. The plot illustrates the mean ranks (vertical axis) calculated from the rank given by respondents to each factor listed on the horizontal axis. LN, lymph node.

Of the eight factors illustrated in Figure 1, T staging alone, the size of LN metastases, and the location of LN metastases were rated overall as the top three factors determining resectability. Specifically, surgeons placed the most importance on the location of LN metastases (mean rank = 3.2), whereas oncologists ranked T staging alone as the most important factor (mean rank = 1.8).

Interlobular N1, hilar N1, and single-station N2 disease were considered resectable by all respondents (100%). Lower mediastinal N2 and upper mediastinal N2 disease were considered resectable by 91.7% and 78.3% of respondents, respectively. Overall, 70.0% of respondents considered T4 N0, T3 N1, and T4 N1 to be distinct in terms of treatment options, although opinions differed substantially between surgeons and oncologists. A significantly higher proportion of oncologists versus surgeons (95.8% and 52.8%, respectively) would consider and treat T4 N0, T3 N1, and T4 N1 as distinct (*p* < 0.001). The definition of bulky N2 disease also tended to differ between surgeons and oncologists (*p* < 0.05). Among oncologists, 91.7% defined bulky disease as N2 nodal involvement with a minimal greatest dimension of greater than 3 cm, compared with 58.3% of surgeons using this definition; 36.1% of surgeons, instead, defined bulky disease as nodal involvement with a minimal greatest dimension of 2 cm.

### Resectability as Defined Using Case Scenarios

**Figure 2:**
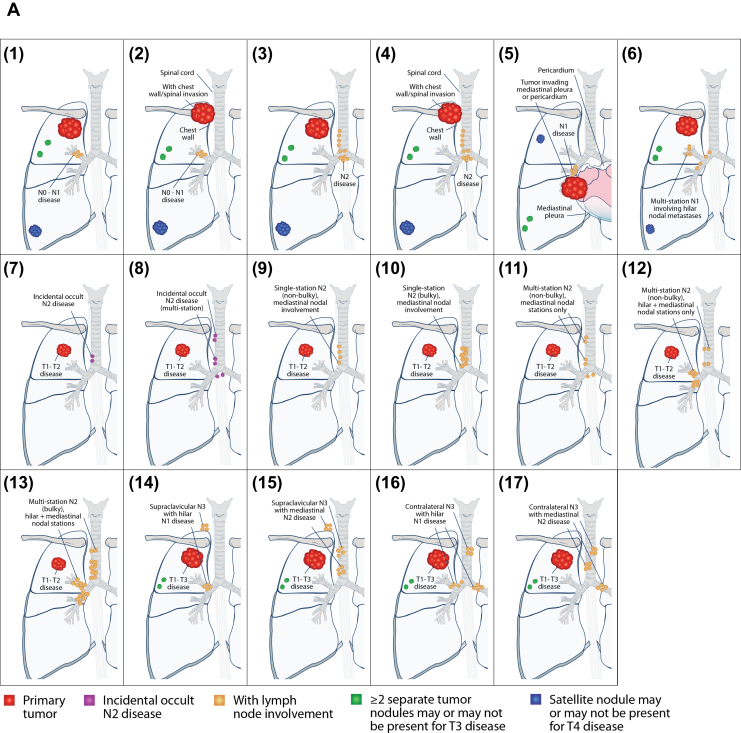

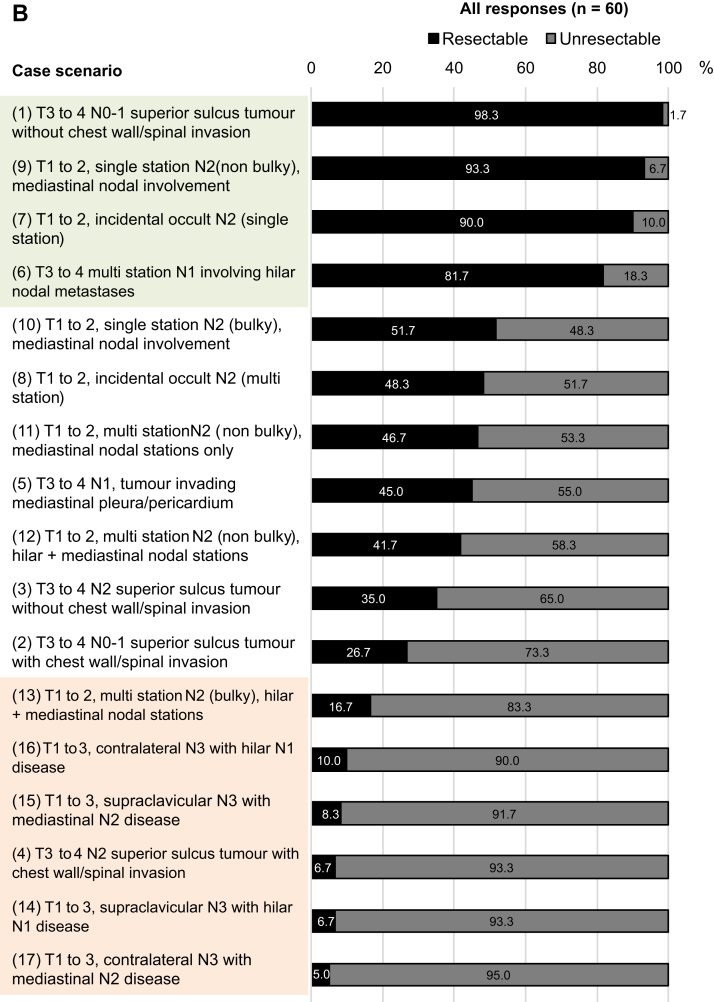
Stage III NSCLC case scenarios and assessment of resectability. (*A*) Anatomical illustrations corresponding to 17 case scenarios described in the survey. These represent a range of “potentially resectable” scenarios (with or without other preoperative treatment), for which respondents were asked to indicate their views on potential resectability and appropriate treatments. Tumor and node stage descriptions are based on the eighth edition of the AJCC/UICC TNM staging system for lung cancer: (1) T3 to 4 N0-1 superior sulcus tumor without chest wall/spinal invasion; (2) T3 to 4 N0-1 superior sulcus tumor with chest wall/spinal invasion; (3) T3 to 4 N2 superior sulcus tumor without chest wall/spinal invasion; (4) T3 to 4 N2 superior sulcus tumor with chest wall/spinal invasion; (5) T3 to 4 N1, tumor invading mediastinal pleura/pericardium; (6) T3 to 4 multi-station N1 involving hilar nodal metastases; (7) T1 to 2, incidental occult N2 (single-station)∗; (8) T1 to 2, incidental occult N2 (multi-station)∗; (9) T1 to 2, single station N2 (non-bulky), mediastinal nodal involvement; (10) T1 to 2, single station N2 (bulky), mediastinal nodal involvement; (11) T1 to 2, multistation N2 (nonbulky), mediastinal nodal stations only; (12) T1 to 2, multi-station N2 (nonbulky), hilar plus mediastinal nodal stations; (13) T1 to 2, multi-station N2 (bulky), hilar plus mediastinal nodal stations; (14) T1 to 3, supraclavicular N3 with hilar N1 disease; (15) T1 to 3, supraclavicular N3 with mediastinal N2 disease; (16) T1 to 3, contralateral N3 with hilar N1 disease; and (17) T1 to 3, contralateral N3 with mediastinal N2 disease. ∗Refers to clinical suspicion of incidental N2 disease based on preoperative findings, not limited to discovery at the time of surgical resection. (*B*) Distribution of overall responses to the question “Please assess resectability for this clinical scenario, assuming good performance status” (answer: resectable/unresectable). AJCC/UICC, American Joint Committee on Cancer/Union for International Cancer Control.

Respondents were presented with a set of hypothetical case scenarios describing the range of presentations of stage III NSCLC often seen in practice. These 17 scenarios are illustrated in Figure 2*A*. On the basis of the scenario descriptions, and assuming good PS, respondents were asked to rate each scenario as resectable or unresectable (Fig. 2*B*). A threshold of 80% was interpreted as an agreement among respondents regarding the resectability of each scenario. Overall, four scenarios were considered resectable and six were considered unresectable by at least 80% of respondents. The four resectable scenarios had the following features: (1) limited extent of LN involvement (e.g., hilar nodes only or single-station); (2) nonbulky disease; or (3) absence of chest wall or spinal invasion. In contrast, the six unresectable scenarios featured extensive nodal involvement (e.g., N3 disease with involvement of contralateral or supraclavicular nodes, multistation N2 disease), bulky disease, or presence of chest wall or spinal invasion.

The remaining seven scenarios were associated with lower overall levels of agreement on resectability (Fig. 2*B*). These “borderline” scenarios represent certain combinations of factors mentioned above (Fig. 1) that make an assessment of resectability more complex and subjective, which includes lower T stage but with bulky N2 disease or incidental occult multistation N2 tumors invading mediastinal pleura or pericardium, or hilar and mediastinal nodal involvement. Resectability assessments for superior sulcus tumors were heterogeneous in different scenarios: T3 to 4 N0-1 without chest wall invasion (scenario 1) was considered resectable (98.3%), whereas T3 to 4 N2 superior sulcus tumor with chest wall/spinal invasion (scenario 4) was considered unresectable by 93.3% of respondents. Agreement on resectability was also low for T3 to 4 N2 tumors without chest wall/spinal invasion (scenario 3) (35.0% agreed this was resectable), and T3 to 4 N0-1 tumors with chest wall/spinal invasion (scenario 2) (26.7% agreed this was resectable).

**Figure 3:**
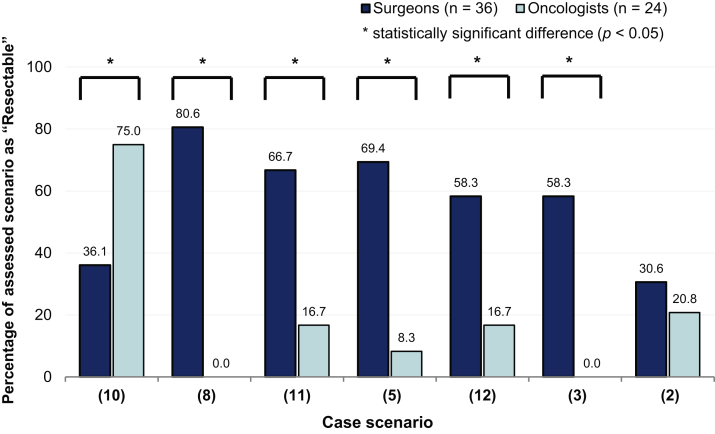
Surgeons’ and oncologists’ assessment of resectability in borderline scenarios (<80% overall agreement on resectability). Distribution of surgeons’ and oncologists’ responses to the question “Please assess resectability for this clinical scenario, assuming good performance status” (answer: resectable/unresectable). Scenarios illustrated are the following: (2) T3 to 4 N0-1 superior sulcus tumor with chest wall/spinal invasion; (3) T3 to 4 N2 superior sulcus tumor without chest wall/spinal invasion; (5) T3 to 4 N1, tumor invading mediastinal pleura/pericardium; (8) T1 to 2, incidental occult N2 (multistation)∗∗; (10) T1 to 2, single station N2 (bulky), mediastinal nodal involvement; (11) T1 to 2, multistation N2 (nonbulky), mediastinal nodal stations only; and (12) T1 to 2, multistation N2 (nonbulky), hilar plus mediastinal nodal stations. ∗Represents statistically significant difference between surgeons and oncologists (*p* < 0.05). ∗∗Refers to clinical suspicion of incidental N2 disease based on preoperative findings, not limited to discovery at the time of surgical resection.

For six of the scenarios with lower agreement on resectability, surgeons’ and oncologists’ responses differed significantly (Fig. 3). In most cases, a higher percentage of surgeons than oncologists considered the scenario resectable. The exception was T1 to 2, single-station N2 (bulky) with mediastinal nodal involvement (scenario 10), which 75.0% of oncologists considered resectable, compared with only 36.1% of surgeons. There were two scenarios—T1 to 2, incidental occult N2 (multistation) (scenario 8) and T3 to 4 N2 superior sulcus tumor without chest wall/spinal invasion (scenario 3)—which 100% of oncologists considered unresectable, in contrast to 81% and 58% of surgeons who considered these resectable, respectively.

### Patient Selection and Treatment Paradigms for Stage III NSCLC

For each of the 17 scenarios presented, respondents were asked to indicate their preferred treatment options. For the four resectable scenarios, all respondents indicated they would consider neoadjuvant therapy (usually chemotherapy or CRT) before surgery; greater than or equal to 90% of respondents indicated they would consider adjuvant therapy, generally cCRT or chemotherapy (Supplementary Table 3). Respondents would also consider targeted therapy (61.7%) when actionable mutations were present, or immunotherapy (66.7%) as adjuvant treatment after surgery. Across the six unresectable scenarios, the preferred options were cCRT followed by consolidation immunotherapy in most cases, or induction chemotherapy followed by cCRT (Supplementary Table 4). The preferred treatment options for borderline scenarios are presented in Supplementary Table 5. Among oncologists, 66.7% would consider cCRT for patients aged up to 85 years (Supplementary Fig. 3*A*). Most respondents selected platinum-based chemotherapy and pemetrexed for definitive cCRT (Supplementary Fig. 3*B*). Most oncologists would consider surgery after definitive cCRT when there was good radiologic response but mediastinoscopy had positive results (87.5%, *p* < 0.001), whereas surgeons would consider surgery when stable disease was achieved with cCRT (63.9%, *p* < 0.001), or with a good radiologic response and either negative mediastinoscopy (69.4%, *p* < 0.001) or mediastinoscopy were not done (58.3%, *p* = 0.007) (Supplementary Fig. 3*C*). Overall, CT scan (85.0%) and brain magnetic resonance imaging (73.3%) were the most common modalities for follow-up after definitive CRT.

### Multidisciplinary Discussion of Stage III NSCLC in People’s Republic of China, Hong Kong, and Macau

**Figure 4:**
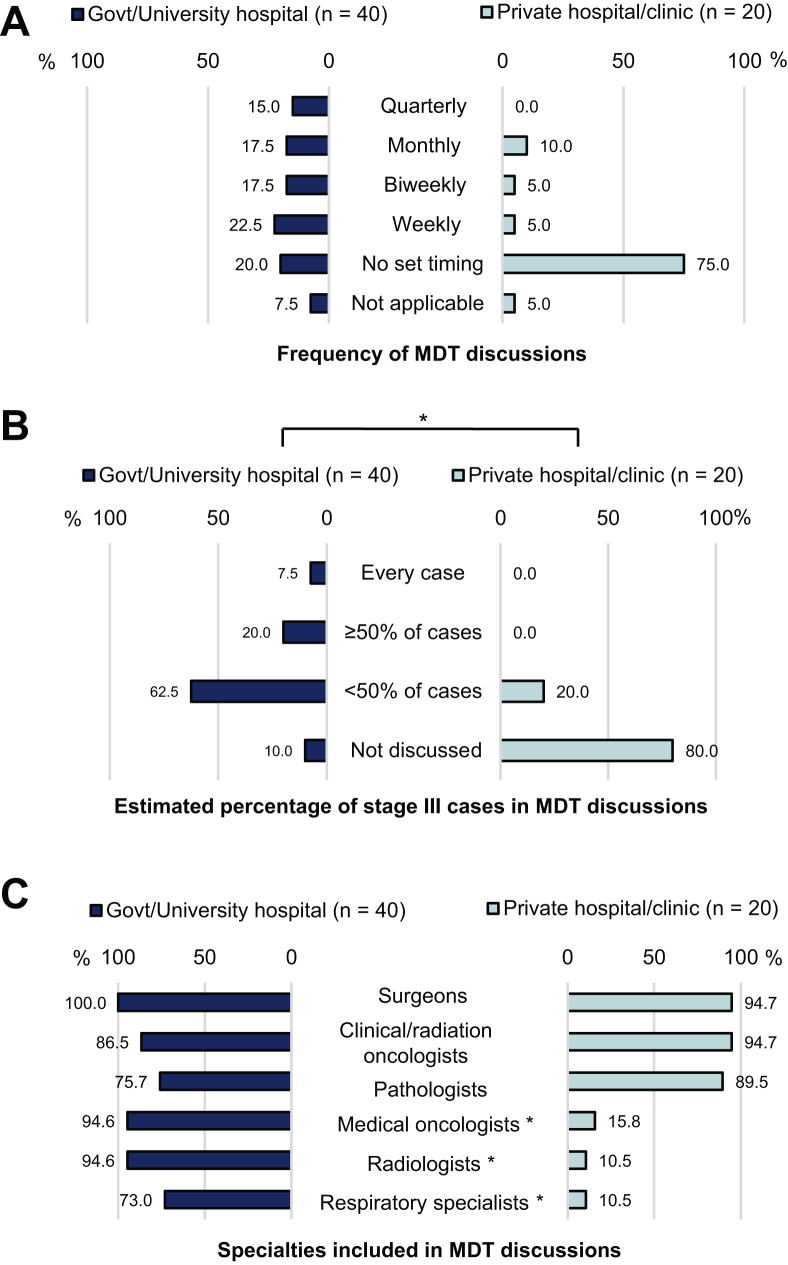
The current implementation of MDT discussions in the People’s Republic of China, Hong Kong, and Macau. (*A*) Frequency of MDT discussions. (*B*) Estimated percentage of stage III cases discussed in MDT discussions. (*C*) Specialties included in MDT discussions. ∗Represents statistically significant difference between those working in government/university hospitals and private hospitals/clinics (*p* < 0.05). Govt, government; MDT, multidisciplinary team.

Most respondents had access to MDT discussions in either traditional or digital/online format, with only 11.7% reporting no MDT discussions in their practice. Half of the respondents (50.0%) had participated in MDT discussions on digital/online platforms. A significantly higher percentage of respondents in public institutions (67.5%) had participated in digital/online MDT discussions compared with those in private hospitals/clinics (15.0%) (*p* < 0.001). Overall, 55.0% of respondents indicated that MDT discussions were convened at regular intervals, ranging from weekly to monthly or quarterly. Having MDT discussions at regular intervals was more often reported by those in public institutions (72.5%) than by those in private hospitals/clinics (20.0%) (Fig. 4*A*).

Although two-thirds of respondents indicated stage III cases were discussed in MDT discussions (in-person or digital/online), only selected stage III cases (<50%) were discussed in a multidisciplinary setting across public and private institutions. Most participants in private institutions (80.0%) did not discuss stage III cases in MDT discussions; the rest indicated that only selected stage III cases were discussed (Fig. 4*B*). In public institutions, only 7.5% of respondents reported that every stage III case was discussed, 20.0% reported that at least 50% of cases were discussed, and 62.5% discussed only selected cases. MDT discussions usually included surgeons (98.2%), clinical/radiation oncologists (89.3%), and pathologists (80.4%), less often medical oncologists (67.9%), radiologists (66.1%), and respiratory specialists (51.8%). Medical oncologists, radiologists, and respiratory specialists were included significantly more often in public institutions than in private institutions (*p* < 0.001 for all) (Fig. 4*C*).

## Discussion

This survey of lung cancer specialists in the People’s Republic of China, Hong Kong, and Macau indicates the broad alignment of practice with current guideline recommendations,^14^^,^^17^^,^^19^ although heterogeneity was apparent, most notably in the specialists’ assessment of resectability. We have, therefore, focused on the issues of resectability and multidisciplinary discussion for stage III NSCLC here. The survey also yielded information on diagnosis/staging practice and prevailing treatment approaches for resectable and unresectable disease, and these could be taken as a starting point to inform personalized management.

A major strength of this survey is the use of a comprehensive set of case scenarios to understand in detail in which surgeons’ and oncologists’ working concepts of resectability are similar and when they may differ. Although respondents agreed on major features of the resectable disease, and clearly identified 11 of 17 case scenarios as resectable or unresectable, there was clearly heterogeneity in their views on “borderline” scenarios, reflecting real-life uncertainty or subjectivity. The heterogeneity might also be related to the current lack of unified criteria for resectability and potential ambiguity in some areas of existing guidelines.^5^^,^^6^ For example, a comparative analysis of simplified treatment decision criteria for stage III N2 NSCLC from various guidelines highlighted nonbulky multistation N2 disease as one area of greater variation in recommendations,^5^ with around half recommending surgery-based approaches, and the rest indicating no preference or radiotherapy-based approaches. Similarly, in our survey, we observed minimal overall agreement between surgeons and oncologists on resectability of nonbulky multistation N2 with extensive nodal involvement (scenarios 11 and 12).

**Figure 5:**
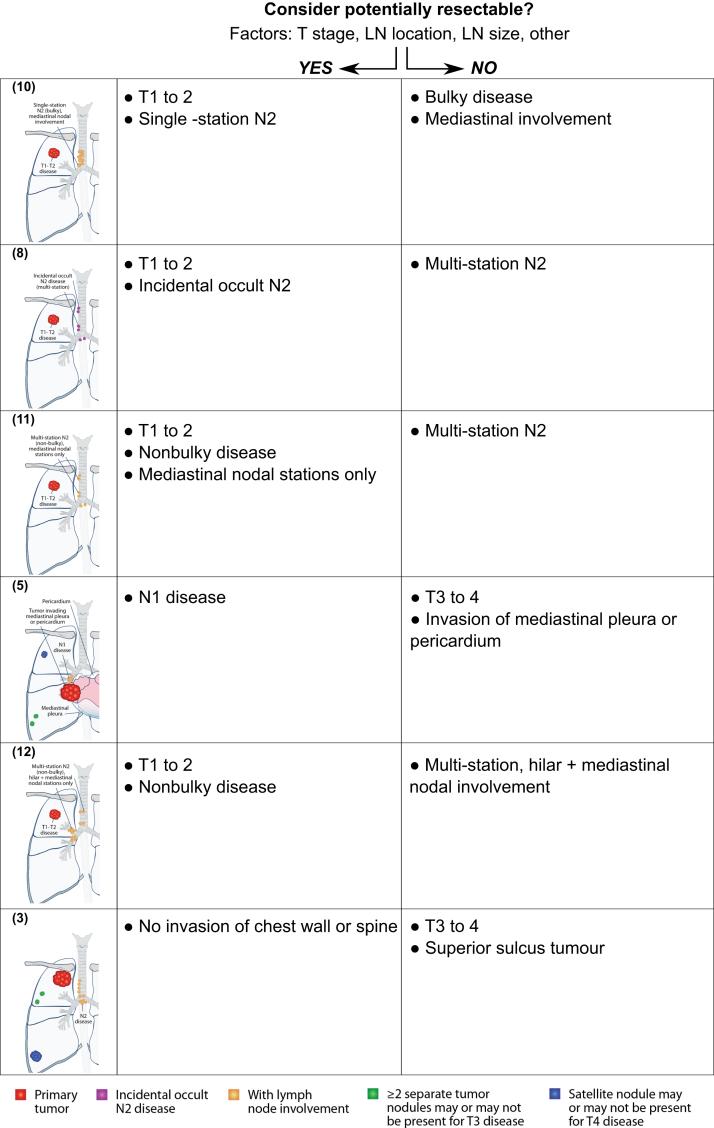
Considerations in balancing multiple factors that determine resectability. Scenarios illustrated are the following: (3) T3 to 4 N2 superior sulcus tumor without chest wall/spinal invasion; (5) T3-4 N1, tumor invading mediastinal pleura/pericardium; (8) T1 to 2, incidental occult N2 (multistation)∗; (10) T1 to 2, single station N2 (bulky), mediastinal nodal involvement; (11) T1 to 2, multi-station N2 (nonbulky), mediastinal nodal stations only; and (12) T1 to 2, multistation N2 (non-bulky), hilar plus mediastinal nodal stations. ∗Refers to clinical suspicion of incidental N2 disease based on preoperative findings, not limited to discovery at the time of surgical resection. LN, lymph node.

In this survey, we realized that oncologists and surgeons may evaluate resectability from slightly different perspectives. In Figure 5, we consider specific features of the borderline case scenarios presented in the survey. Each of these had multiple features that could either favor or contraindicate the possibility of surgical resection, depending on a specialist’s judgment. In general, oncologists may be more accustomed to reviewing CT images at axial cuts and, thus, tend to perceive resectability from a two-dimensional perspective. In contrast, surgeons are trained to appraise resectability from a three-dimensional and spatial perspective besides assessing the technical difficulty of operations. This tactile sense, reinforced through experience gained over many operations, may lead them to determine resectability differently from oncologists. This may explain why surgeons considered five clinical scenarios (scenarios 8, 11, 5, 12, and 3) resectable. In contrast, bulky disease, albeit involving only one nodal station (scenario 10), may be perceived by surgeons as posing significant operative difficulty and precluding gross tumor removal, but was considered resectable by oncologists. This clearly illustrates that MDT discussions are an essential platform to resolve such discrepancies in perceiving resectability.

MDT discussion has exhibited the potential to improve disease staging, influence treatment plans, and increase adherence to care guidelines.^33^^,^^34^ In a recent study, OS was found to be longer for patients with stage III NSCLC treated after MDT discussion than those treated without MDT; moreover, MDT discussion was identified as an independent prognostic factor along with PS and surgical resection.^29^ To obtain the full benefit of the MDT process, a range of specialties should be represented and, if possible, all stage III NSCLC cases should be discussed at various stages of treatment. In our view, such consensus among specialists should ideally be an ongoing process as this allows the care team opportunities to reassess and revise the treatment plan given that additional clinical, radiologic, and histologic findings emerge especially after induction treatment. Furthermore, the conclusions and rationale for the MDT treatment recommendations should be conveyed to the patient in a timely and accurate way to allow them to make informed decisions.

However, our results indicate that only selected stage III cases are currently discussed in MDT meetings in respondents’ institutions. With potentially limited resources, it is highly desirable to have a practical and systematic approach to help specialists prioritize cases for multidisciplinary discussion, even if not all stage III NSCLC cases can be discussed in a formal MDT meeting. The insights from the case scenarios may be directly applicable in this regard. The existing scenario descriptions were made as comprehensive as possible under the constraints of the survey setting; however, we acknowledge that they are not exhaustive and variability in the interpretation of scenarios is possible. In the future, if these clinical scenarios are to be used outside of the original survey context as a reference resource or for developing a multidisciplinary consensus on early-stage NSCLC management, it will be important to include additional context and sufficient explanation for users.

The results of this survey should be interpreted in light of certain limitations. First, this survey obtained responses from 60 respondents, mainly from South China, Hong Kong, and Macau, thus, representing a small fraction of all Chinese lung cancer specialists. As with surveys involving a convenience sample, the potential effects of sampling bias need to be considered. Further initiatives are needed to ascertain how well the findings reflect general trends in lung cancer management in the People’s Republic of China. As the first study of this kind in our region (to our knowledge), it may be premature to comment on the potential generalizability of our observations to other regions, especially where routine MDT implementation is expected to be more prevalent as in a number of European countries and Australia.^35^, ^36^, ^37^ Nevertheless, we would still expect some differences in specialists’ perspectives that are best resolved within an MDT meeting or equivalent process. In addition, there could be variations related to other characteristics such as center case volume, MDT panel composition, or access to specialist diagnostic facilities and expertise.^38^^,^^39^ Although we were unable to explore these aspects using our data set, we welcome further research in settings or regions where MDTs are more prevalent, as this could provide interesting contrasts and insights into how best to use MDTs across a range of resource level settings. For example, it is highly recommended for patients with suspected N2 disease to undergo more comprehensive imaging and histologic investigations with PET-CT and EBUS-guided TBNA, among others, to better delineate their nodal status. However, within the People’s Republic of China, PET-CT and EBUS are available but are generally not reimbursed for use in stage III NSCLC. With EBUS, the availability of practitioners with proficiency in the technique is another practical limitation. Therefore, MDT discussion may be especially beneficial in such cases to guide diagnostic and treatment decisions on the basis of available resources.

We note that the treatment landscape for NSCLC continues to evolve rapidly; thus, with new and potentially practice-changing evidence available, some trends captured in this survey may be less reflective of current treatment paradigms. For example, this survey was conducted from May 2020 to August 2020, before the approval of osimertinib as adjuvant treatment for patients with resected EGFR-mutated NSCLC and additional evidence for immunotherapies, such as the CheckMate 816 study, emerged. With such evidence available, we perceive that more respondents would consider targeted therapies such as osimertinib and immunotherapies in the adjuvant and neoadjuvant settings.

This survey highlights the need for clearer definitions of potential resectability to facilitate rapid review and appropriate selection of patients for multimodality therapy. This issue is likely to become more prominent as additional treatment options for stage III NSCLC continue to emerge. With limited resources yet increasingly diverse and complex treatment options for care teams to consider, it will be important to strategically and efficiently prioritize stage III NSCLC cases that require MDT discussion. In tandem, the use of digital technologies may facilitate easier access to MDT participation. The survey indicates that respondents are already familiar with using online platforms to facilitate formal or informal discussions, likely accelerated by the need to adapt to restrictions in the midst of the ongoing coronavirus disease 2019 pandemic. The mass adoption of digital technologies, especially in Asia, and improvements in telemedicine solutions make it feasible to transition to hybrid or entirely online MDTs.^40^ For example, The University of Hong Kong-Shenzhen Hospital currently accepts referrals to provide a remote review for patients from anywhere in the People’s Republic of China. Such initiatives could potentially broaden access to multidisciplinary expertise for clinicians practicing outside of high-volume expert centers, and benefit patients living in remote rural areas.

In conclusion, our findings indicate variability between surgeons and oncologists in terms of working definitions and assessment of resectability, as illustrated on a comprehensive set of 17 stage III NSCLC case scenarios. Through these, we have identified types of cases associated with diverging opinions among specialists, representing clinical scenarios for which more comprehensive multidisciplinary discussion may be needed. Our other findings on prevailing treatment approaches for resectable and unresectable diseases could be taken as a starting point to inform personalized therapy selection. As implementation of multidisciplinary review for stage III NSCLC cases seems variable across Chinese institutions, specialists could use these insights to prioritize their stage III cases for multidisciplinary discussions and maximize limited local resources. At the same time, it would be valuable to develop a multidisciplinary expert consensus statement to harmonize treatment approaches in NSCLC. This would complement local institutional expertise toward improving real-world decision-making and personalized disease management in NSCLC.

## CRediT Authorship Contribution Statement

**Victor Ho-Fun Lee:** Conceptualization, Methodology, Investigation, Data curation, Project administration, Writing - review & editing.

**Joseph Siu Kie Au:** Conceptualization, Methodology, Investigation, Data curation, Project administration, Writing - review & editing.

**Ju-Wei Mu, Guangli Xiao, Fiona Mei Ying Lim, Hon Chi Suen, Kam Hung Wong:** Investigation, Writing - review & editing.
