# Supplementary material for: Real-World Perspectives From Surgeons and Oncologists on Resectability Definition and Multidisciplinary Team Discussion of Stage III NSCLC in People’s Republic of China, Hong Kong, and Macau: A Physician Survey
Source: JTO Clin Res Rep. 2022 Mar 19;3(5):100308. doi: 10.1016/j.jtocrr.2022.100308 (PMC9011118; doi:10.1016/j.jtocrr.2022.100308)
Supplement: Supplementary material [file mmc1.docx]

# Supplementary Figures

## Supplementary Figure 1. Typical stage III NSCLC presentation in respondents’ clinical practice

A. Most common stage presentation for stage III NSCLC cases seen in clinical practice. B. Percentage of stage III cases considered resectable. C. Percentage of stage III cases staged as N2. D. Percentage of stage III N2 cases receiving surgery. N2 refers to ipsilateral and/or subcarinal mediastinal lymph node involvement, as defined in the 8th edition of the American Joint Committee on Cancer/Union for International Cancer Control (AJCC/UICC) tumor-node-metastasis (TNM) staging system for lung cancer. * Indicates statistically significant difference between surgeons and oncologists (p<0.05).


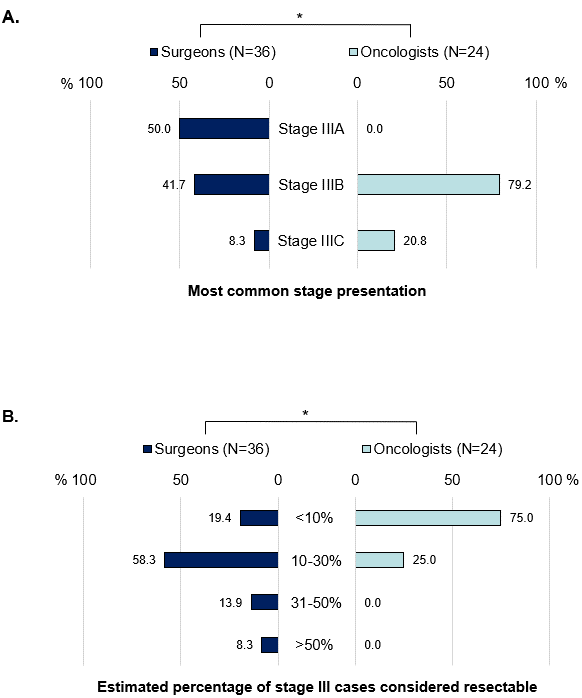

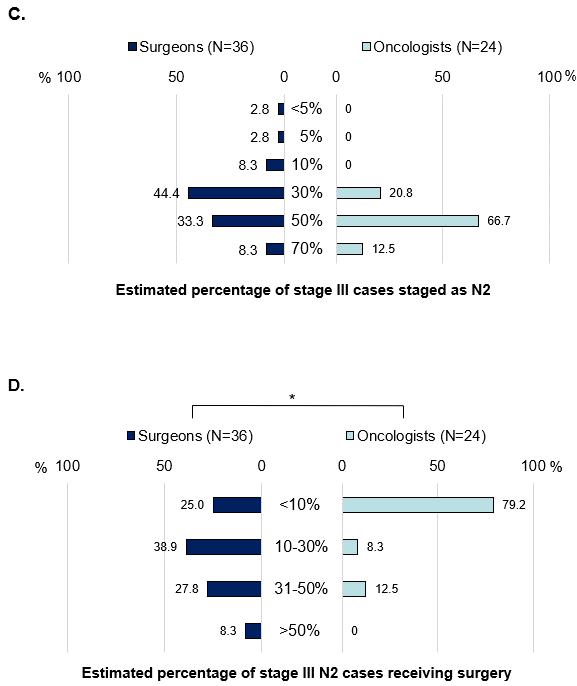


## Supplementary Figure 2. Diagnostic tests routinely used for stage III NSCLC

* indicates statistically significant difference between surgeons and oncologists (p<0.05). MRI, magnetic resonance imaging; CT, computed tomography; TBNA, transbronchial needle aspiration; EBUS TBNA, endobronchial ultrasound-guided TBNA; EUS FNA, endoscopic ultrasound-guided fine-needle aspiration.


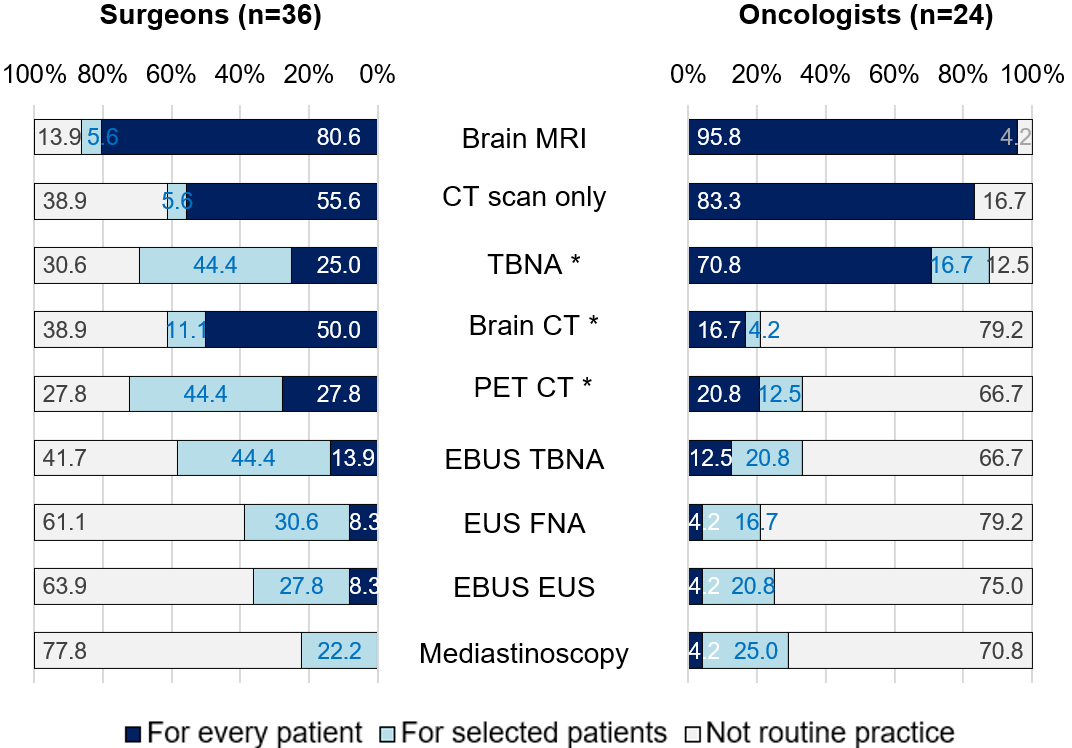


## Supplementary Figure 3. Preferred options for definitive chemoradiation

A. Age limit for concurrent CRT. B. Preferred chemotherapy for concurrent CRT. C. Role for curative surgery after definitive CRT. * indicates statistically significant difference between surgeons and oncologists (p<0.05). CRT, chemoradiotherapy


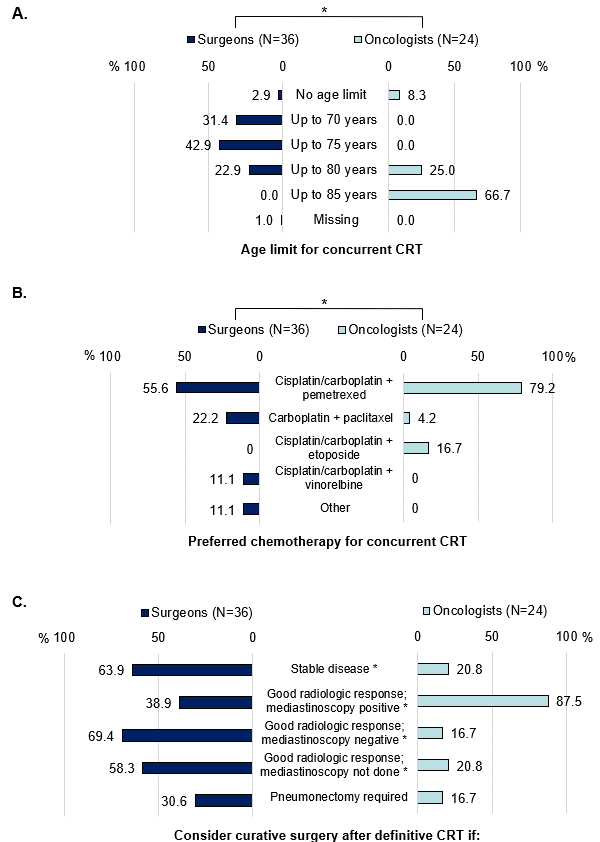


# Supplementary Tables

## Supplementary Table 1: Survey Questionnaire

| **Question** | | **Response options** |
| --- | --- | --- |
| **Section 0: Demographics** | | |
| 1. | Please indicate your specialty | 1. Surgeon |
|  |  | 1. Medical Oncologist |
|  |  | 1. Radiation Oncologist |
|  |  | 1. Clinical Oncologist |
|  |  | 1. Respiratory Physicians / Pulmonologists |
| 2. | Please indicate your year of service in your specialty | 1. 1-2 years |
|  |  | 1. 3-5 years |
|  |  | 1. 6-10 years |
|  |  | 1. 11-20 years |
|  |  | 1. >20 years |
| 3. | Please advise your region of clinical practice | 1. Hong Kong |
|  |  | 1. Macau |
|  |  | 1. North China |
|  |  | 1. South China |
| 4. | What is your primary working institution? | 1. Government hospital |
|  |  | 1. University teaching hospital |
|  |  | 1. Private hospital |
|  |  | 1. Private clinic |
| **Section 1: Staging practice** | | |
| 5. | How often do you see stage III NSCLC? | 1. <10 cases per month |
|  |  | 1. 10-30 cases per month |
|  |  | 1. 31-50 cases per month |
|  |  | 1. >50 cases per month |
| 6. | Among stage III NSCLC, what is the most common stage presentation (based on the 8^th^ edition of AJCC/UICC Staging Classification) in your clinic? | 1. Stage IIIA |
|  |  | 1. Stage IIIB |
|  |  | 1. Stage IIIC |
| 7. | Is there a multi-disciplinary tumor board (MDT) in your clinical practice, and are you involved in the MDT meeting? | 1. Yes, there is a MDT meeting and I am involved in MDT meeting |
|  |  | 1. Yes, there is a MDT meeting but I am not involved |
|  |  | 1. No MDT meeting |
| 8. | How often is the MDT? | 1. Quarterly / Bimonthly |
|  |  | 1. Monthly |
|  |  | 1. Biweekly |
|  |  | 1. Weekly |
|  |  | 1. No set timing |
|  |  | 1. Not applicable |
| 9. | Are you involved in MDT discussions in digital or social media platforms (including phone calls, whatsapp/wechat group, or any other online platform)? | 1. Yes |
|  |  | 1. No |
| 10. | Would you discuss stage III cases in a multi-disciplinary setting?  (applicable to MDT meeting or MDT discussions through social media/phone calls) | 1. Every stage III NSCLC case |
|  |  | 1. >=50% stage III NSCLC cases |
|  |  | 1. Selected stage III NSCLC cases (<50%) |
|  |  | 1. No |
| 11. | What are the specialties involved in your MDT discussion for stage III NSCLC?  (applicable to MDT meeting or MDT discussions through social media/phone calls)  (Check all appropriate answers.) | 1. Respiratory specialists |
|  |  | 1. Pathologists |
|  |  | 1. Surgeons |
|  |  | 1. Clinical Oncologists / Radiation Oncologists |
|  |  | 1. Radiologists |
|  |  | 1. Medical Oncologists |
|  |  | 1. Not applicable |
| 12. | Please specify if the following diagnostic tests are your usual clinical practice in staging stage III NSCLC cases. | 1. Yes, for every patient |
|  | 1. PET-CT scanning | 1. Yes, for selected patients (please specify) |
|  | 1. EBUS-TBNA | 1. No, not routine practice |
|  | 1. Brain MRI |  |
|  | 1. Brain CT |  |
|  | 1. CT-scan only |  |
|  | 1. Mediastinoscopy |  |
|  | 1. TBNA |  |
|  | 1. EUS-NA |  |
|  | 1. EBUS-EUS |  |
| 13. | Is pathologic staging your routine practice to confirm Stage III NSCLC? | 1. Yes |
|  |  | 1. No |
| 14. | Would you order or perform the following genetic/mutation tests at initial diagnostic workup for stage III NSCLC patients?  (Check all appropriate answers which apply) | 1. EGFR |
|  |  | 1. ALK |
|  |  | 1. ROS1 |
|  |  | 1. KRAS |
|  |  | 1. PD-L1 |
|  |  | 1. A panel (e.g. 5-10 biomarkers) of mutation tests |
|  |  | 1. NGS |
|  |  | 1. No biomarker testing |
| **Section 2: Resectability** | | |
| 15. | How many stage III patients presented at your practice are considered resectable? | 1. 10% |
|  |  | 1. 10-30% |
|  |  | 1. 30-50% |
|  |  | 1. >50% |
| 16. | Rate the following factors in determining resectability at stage III setting? (1 as most important, 8 as least important) | 1. T staging alone |
|  |  | 1. Location of lymph node metastases |
|  |  | 1. Need for pneumonectomy |
|  |  | 1. Performance status |
|  |  | 1. Comorbidities |
|  |  | 1. Lung function |
|  |  | 1. Histology (squamous / non-squamous) |
|  |  | 1. Size of lymph node metastases |
| 17. | Among stage III patients presented at your clinic, how many are staged as N2? | 1. <5% |
|  |  | 1. 5% |
|  |  | 1. 10% |
|  |  | 1. 30% |
|  |  | 1. 50% |
|  |  | 1. 70% |
| 18. | Among stage III, N2 patients presented at your clinic, how many patients will go into surgery? | 1. <10% |
|  |  | 1. 10-30% |
|  |  | 1. 30-50% |
|  |  | 1. >50% |
| 19. | Among patients with resectable stage 3 NSCLC at your clinic, how many of them require pneumonectomy? | 1. 0% |
|  |  | 1. <10% |
|  |  | 1. 10-30% |
|  |  | 1. 30-50% |
|  |  | 1. >50% |
| 20. | Among stage IIIA, do you consider T4 N0, T3 N1 and T4 N1 as single disease with same treatment option? | 1. Yes |
|  |  | 1. No |
| 21. | Please advise resectability in the following lymph node locations | 1. Resectable |
|  | 1. Interlobular N1 | 1. Unresectable |
|  | 1. Hilar N1 |  |
|  | 1. Single station N2 |  |
|  | 1. Upper mediastinal N2 |  |
|  | 1. Lower mediastinal N2 |  |
| 22. | How would you define bulky N2 disease? | 1. N2 nodal involvement with minimal greatest dimension of 2cm |
|  |  | 1. N2 nodal involvement with minimal greatest dimension >3cm |
|  |  | 1. Other |
| **Section 3: Treatment paradigm** | | |
| 23. | Would you consider targeted therapy a treatment option for stage III NSCLC? (Check all appropriate answers which apply) | 1. Yes, as neoadjuvant treatment before surgery |
|  |  | 1. Yes, as adjuvant treatment after surgery |
|  |  | 1. Yes, as induction therapy before definitive CRT |
|  |  | 1. Yes, as consolidation therapy after definitive CRT in unresectable patients |
|  |  | 1. Yes, as palliative treatment when radical treatment is not feasible and actionable mutation is detected. |
|  |  | 1. No, targeted therapies are not a treatment option in stage III NSCLC |
| 24 | Would you consider immunotherapy (with immune checkpoint inhibitors) alone, or immunotherapy with chemotherapy a treatment option for stage III NSCLC?  (Check all appropriate answers which apply) | 1. Yes, neoadjuvant treatment before surgery |
|  |  | 1. Yes, as adjuvant treatment after surgery |
|  |  | 1. Yes, as palliative treatment when radical treatment is not feasible and actionable mutation is detected. |
|  |  | 1. No, immunotherapy is not a treatment option in stage III NSCLC |
| 25. | Are there any of your patients treated with curative surgery for residual tumor after definitive concurrent CRT? | 1. <10% |
|  |  | 1. 10-30% |
|  |  | 1. 30-50% |
|  |  | 1. >50% |
|  |  | 1. No |
| 26. | Would you consider curative surgery after concomitant chemoradiation in the following condition? | 1. Yes 2. No |
|  | 1. Stable disease following chemoradiation |  |
|  | 1. Good response after chemoradiation radiologically, but mediastinoscopy positive after chemoradiation |  |
|  | 1. Good response radiologically after chemoradiation, mediastinoscopy negative after chemoradiation |  |
|  | 1. Good response radiologically after chemoradiation, mediastinoscopy not done after chemoradiation |  |
|  | 1. Pneumonectomy would be required |  |
| 27. | What is your preferred chemotherapy for definitive chemoradiation? | 1. Cisplatin/carboplatin + vinorelbine |
|  |  | 1. Cisplatin/carboplatin + etoposide |
|  |  | 1. Cisplatin/carboplatin + pemetrexed |
|  |  | 1. Carboplatin + paclitaxel |
|  |  | 1. Other:______________________________ |
| 28. | What is your routine radiation technique in definitive chemoradiation? | 1. IMRT/tomotherapy/VMAT |
|  |  | 1. Conformal 3D RT |
|  |  | 1. Conventional 2D RT |
|  |  | 1. SBRT |
|  |  | 1. Proton therapy or charged particle therapy |
| 29. | Please indicate how many percentage of patients undergoing definitive chemoradiation are treated with the following radiation dose? |  |
|  | (a) 54Gy | 1. ≤10% |
|  | (b) 60Gy | 1. >11-30% |
|  | (c) >60 to 66 Gy | 1. 31-50% |
|  | (d) >66Gy | 1. >50% |
|  |  | 1. I have not prescribed this radiation dose range to stage III patients. |
| 30. | Is there an age above which you would not give concomitant chemoradiation for stage III NSCLC | 1. No |
|  |  | 1. Yes, 70 is the limit |
|  |  | 1. Yes, 75 is the limit |
|  |  | 1. Yes, 80 is the limit |
|  |  | 1. Yes, 85 is the limit |
|  |  | 1. Yes, other:_________________________ |
| 31. | Is there a subset of stage III patient you would consider palliative treatment (treat as stage 4)? | 1. Yes, <10% |
|  |  | 1. Yes, 10-30% |
|  |  | 1. Yes, 31-50% |
|  |  | 1. Yes, >50% |
|  |  | 1. No |
| 32. | What is your routine follow up procedure after definitive chemoradiation?  (check all appropriate answers)  Please indicate frequency of follow up procedure. | 1. CT-PET   Frequency:_______________________ |
|  |  | 1. CT scan   Frequency:_________________________ |
|  |  | 1. Brain MRI   Frequency:__________________________ |
|  |  | 1. Brain CT   Frequency:__________________________ |
|  |  | 1. Other:____________________________   Frequency:___________________________ |
| 33. | Is this different from routine follow up procedure after surgery in resectable patients? | 1. Yes,   please indicate option a-e and frequency:__________ |
|  |  | 1. No |
| **Section 4: Case Scenarios**  **Answer parts a, b, c, d (below) for the following case scenarios, assuming good PS,** | | |
|  | (1) T3-4 N0-1 superior sulcus tumor without chest wall/spinal invasion | |
|  | (2) T3-4 N0-1 superior sulcus tumor with chest wall/spinal invasion | |
|  | (3) T3-4 N2 superior sulcus tumor without chest wall/spinal invasion | |
|  | (4) T3-4 N2 superior sulcus tumor with chest wall/spinal invasion | |
|  | (5) T3-4 N1, tumor invading mediastinal pleura / pericardium | |
|  | (6) T3-4 multi-station N1 involving hilar nodal metastases | |
|  | (7) T1-2, incidental occult N2 (single-station) | |
|  | (8) T1-2, incidental occult N2 (multi-station) | |
|  | (9) T1-2, single station N2 (non-bulky), mediastinal nodal involvement | |
|  | (10) T1-2, single station N2 (bulky), mediastinal nodal involvement | |
|  | (11) T1-2, multi-station N2 (non-bulky), mediastinal nodal stations only | |
|  | (12) T1-2, multi-station N2 (non-bulky), hilar + mediastinal nodal stations | |
|  | (13) T1-2, multi-station N2 (bulky), hilar + mediastinal nodal stations | |
|  | (14) T1-3, supraclavicular N3 with hilar N1 disease | |
|  | (15) T1-3, supraclavicular N3 with mediastinal N2 disease | |
|  | (16) T1-3, contralateral N3 with hilar N1 disease | |
|  | (17) T1-3, contralateral N3 with mediastinal N2 disease | |
|  | (18) T1-2, multi-station N2 (non-bulky), hilar nodal stations only^a^ | |
| a. | Please advise resectability in this case. | 1. Resectable |
|  |  | 1. Unresectable |
| b. | Would you prescribe neoadjuvant treatment before surgery in this case? | 1. Neoadjuvant chemotherapy |
|  |  | 1. Neoadjuvant chemoradiotherapy |
|  |  | 1. Other neoadjuvant treatment, please specify |
|  |  | 1. No neoadjuvant treatment / not applicable |
| c. | Would you prescribe adjuvant treatment post surgery in this case? | 1. Adjuvant chemotherapy |
|  |  | 1. Adjuvant concurrent chemoradiotherapy |
|  |  | 1. Adjuvant radiotherapy |
|  |  | 1. Adjuvant sequential chemoradiotherapy |
|  |  | 1. Other adjuvant treatment, please specify: |
|  |  | 1. No adjuvant treatments / not applicable |
| d. | If unresectable, please indicate your treatment option for this case. | 1. radical concurrent chemoradiation only |
|  |  | 1. induction chemotherapy followed by concurrent chemoradiation |
|  |  | 1. induction chemotherapy followed by concurrent chemoradiation, followed by consolidation chemotherapy |
|  |  | 1. concurrent chemoradiation followed by consolidation chemotherapy |
|  |  | 1. concurrent chemoradiation followed by consolidation immunotherapy |
|  |  | 1. sequential chemoradiation |
|  |  | 1. sequential chemoradiation followed by consolidation immunotherapy |
|  |  | 1. radiation alone |
|  |  | 1. Other, please specify |

^a^ This question was not analyzed due to ambiguity in the text description of the scenario

## Supplementary Table 2. Use of biomarker testing at initial diagnostic workup

| **Usual clinical practice of survey respondents** | **All responses**  **N=60** |
| --- | --- |
| *EGFR* | 82.1% |
| *ALK* | 82.1% |
| *ROS1* | 76.8% |
| *KRAS* | 66.1% |
| *PD-L1* | 75.0% |
| A panel (e.g. 5-10 biomarkers) of mutation tests | 39.3% |
| NGS | 32.1% |
| Not an option for initial diagnostic workup | 6.7% |

Percentages are based on non-missing responses.

Abbreviations: ALK, anaplastic lymphoma kinase; EGFR, epidermal growth factor receptor; KRAS, kirsten rat sarcoma viral oncogene homolog; PD-L1, programmed death-ligand 1; ROS1, c-ros oncogene 1; NGS, next-generation sequencing.

## Supplementary Table 3. Preferred treatment options for potentially resectable clinical scenarios

| **Clinical scenarios deemed resectable by** ≥**80% of respondents^a^** | **Preferred neoadjuvant treatment**  **N (% of responses)** | **Preferred adjuvant treatment**  **N (% of responses)** |
| --- | --- | --- |
| (1) T3-4 N0-1 superior sulcus tumor without chest wall/spinal invasion | Chemotherapy 33 (55.0) | Chemotherapy 27 (45.0) |
|  | CRT 26 (43.3) | cCRT 25 (41.7) |
|  | Other 1 (1.7) | sCRT 5 (8.3) |
|  |  | Not applicable 3 (5.0) |
| (9) T1-2, single station N2 (non-bulky), mediastinal nodal involvement | Chemotherapy 42 (70.0) | cCRT 29 (48.3) |
|  | CRT 13 (21.7) | Chemotherapy 14 (23.3) |
|  | Other 5 (8.3) | sCRT 12 (20.0) |
|  |  | Radiotherapy 2 (3.3) |
|  |  | Other 2 (3.3) |
|  |  | Not applicable 1 (1.7) |
| (7) T1-2, incidental occult N2 (single-station) | Chemotherapy 37 (61.7) | cCRT 30 (50.0) |
|  | CRT 16 (26.7) | Chemotherapy 19 (31.7) |
|  | Other 7 (11.7) | sCRT 8 (13.3) |
|  |  | Other 2 (3.3) |
|  |  | Not applicable 1 (1.7) |
| (6) T3-4 multi-station N1 involving hilar nodal metastases | Chemotherapy 31 (51.7) | cCRT 33 (55.0) |
|  | CRT 24 (40.0) | Chemotherapy 20 (33.3) |
|  |  | sCRT 6 (10.0) |
|  | Other 5 (8.3) | Radiotherapy 1 (1.7) |

Percentages are based on non-missing responses.

^a^ Based on the response to the question: Please assess resectability for this clinical scenario, assuming good performance status (answer: Resectable / Unresectable)

## Supplementary Table 4. Preferred treatment options for unresectable clinical scenarios

| **Clinical scenarios deemed unresectable by** ≥**80% of respondents^a^** | **Preferred treatment options, N (% of responses)** |
| --- | --- |
| (13) T1-2, multi-station N2 (bulky), hilar + mediastinal nodal stations | cCRT + consolidation immunotherapy 23 (38.3)  Induction chemotherapy + cCRT 19 (31.7)  Induction chemotherapy + cCRT + consolidation chemotherapy 7 (11.7)  cCRT + consolidation chemotherapy 6 (10.0)  Radical cCRT only 2 (3.3)  sCRT + consolidation immunotherapy 1 (1.7)  sCRT 1 (1.7)  Other 1 (1.7) |
| (16) T1-3, contralateral N3 with hilar N1 disease | cCRT + consolidation immunotherapy 23 (38.3)  Induction chemotherapy + cCRT 20 (33.3)  Induction chemotherapy + cCRT + consolidation chemotherapy 6 (10.0)  cCRT + consolidation chemotherapy 3 (5.0)  Radical cCRT only 2 (3.3)  sCRT + consolidation immunotherapy 2 (3.3)  sCRT 1 (1.7)  Other 3 (5.0) |
| (15) T1-3, supraclavicular N3 with mediastinal N2 disease | Induction chemotherapy + cCRT 25 (41.7)  cCRT + consolidation immunotherapy 20 (33.3)  Induction chemotherapy + cCRT + consolidation chemotherapy 3 (5.0)  cCRT + consolidation chemotherapy 4 (6.7)  Radical cCRT only 2 (3.3)  sCRT 2 (3.3)  sCRT + consolidation immunotherapy 1 (1.7)  Radiation alone 1 (1.7)  Other 2 (3.3) |
| (4) T3-4 N2 superior sulcus tumor with chest wall/spinal invasion | cCRT + consolidation immunotherapy 24 (40.0)  Induction chemotherapy + cCRT 19 (31.7)  Induction chemotherapy + cCRT + consolidation chemotherapy 3 (5.0)  cCRT + consolidation chemotherapy 6 (10.0)  Radical cCRT only 5 (8.3)  sCRT + consolidation immunotherapy 2 (3.3)  Radiation alone 1 (1.7) |
| (14) T1-3, supraclavicular N3 with hilar N1 disease | Induction chemotherapy + cCRT 23 (38.3)  cCRT + consolidation immunotherapy 22 (36.7)  Induction chemotherapy + cCRT + consolidation chemotherapy 4 (6.7)  cCRT + consolidation chemotherapy 4 (6.7)  Radical cCRT only 2 (3.3)  sCRT 3 (5.0)  Other 2 (3.3) |
| (17) T1-3, contralateral N3 with mediastinal N2 disease | cCRT + consolidation immunotherapy 22 (36.7)  Induction chemotherapy + cCRT 19 (31.7)  Radical cCRT only 5 (8.3)  Induction chemotherapy + cCRT + consolidation chemotherapy 4 (6.7)  cCRT + consolidation chemotherapy 4 (6.7)  sCRT + consolidation immunotherapy 2 (3.3)  sCRT 1 (1.7)  Other 3 (5.0) |

Percentages are based on non-missing responses.

^a^ Based on the response to the question: Please assess resectability for this clinical scenario, assuming good performance status (response options: Resectable / Unresectable)

## Supplementary Table 5. Preferred treatment options for clinical scenarios representing borderline resectability

| **Clinical scenarios^a^** | **Neoadjuvant treatment**  **N (% of responses)** | **Adjuvant treatment**  **N (% of responses)** | **If unresectable**  **N (% of responses)** |
| --- | --- | --- | --- |
| (10) T1-2, single station N2 (bulky), mediastinal nodal involvement | Chemotherapy 29 (48.3)  CRT 27 (45.0)  Other 4 (6.7) | cCRT 38 (63.3)  sCRT 11 (18.3)  Chemotherapy 6 (10.0)  Radiotherapy 2 (3.3)  Other 1 (1.7)  Not applicable 2 (3.3) | cCRT + consolidation immunotherapy 27 (45.0)  Induction chemotherapy + cCRT 22 (36.7)  Induction chemotherapy + cCRT + consolidation chemotherapy 3 (5.0)  cCRT + consolidation chemotherapy 3 (5.0)  Radical cCRT only 2 (3.3)  sCRT + consolidation immunotherapy 2 (3.3)  sCRT 1 (1.7) |
| (8) T1-2, incidental occult N2 (multi-station) | CRT 30 (50.0)  Chemotherapy 22 (36.7)  Other 8 (13.3) | Chemotherapy 34 (56.7)  cCRT 12 (20.0)  sCRT 9 (15.0)  Radiotherapy 1 (1.7)  Other 1 (1.7)  Not applicable 3 (5.0) | cCRT + consolidation immunotherapy 25 (41.7)  Induction chemotherapy + cCRT 19 (31.7)  Induction chemotherapy + cCRT + consolidation chemotherapy 2 (3.3)  cCRT + consolidation chemotherapy 5 (8.3)  Radical cCRT only 6 (10.0)  sCRT + consolidation immunotherapy 1 (1.7)  sCRT 2 (3.3) |
| (11) T1-2, multi-station N2 (non-bulky), mediastinal nodal stations only | CRT 31 (51.7)  Chemotherapy 24 (40.0)  Other 5 (8.3) | cCRT 32 (53.3)  Chemotherapy 14 (23.3)  sCRT 8 (13.3)  Radiotherapy 2 (3.3)  Other 1 (1.7)  Not applicable 3 (5.0) | cCRT + consolidation immunotherapy 25 (41.7)  Induction chemotherapy + cCRT 23 (38.3)  cCRT + consolidation chemotherapy 5 (8.3)  Induction chemotherapy + cCRT + consolidation chemotherapy 2 (3.3)  Radical cCRT only 2 (3.3)  sCRT 2 (3.3)  sCRT + consolidation immunotherapy 1 (1.7) |
| (5) T3-4 N1, tumor invading mediastinal pleura / pericardium | CRT 34 (56.7)  Chemotherapy 19 (31.7)  Other 7 (11.7) | Chemotherapy 29 (48.3)  cCRT 17 (28.3)  sCRT 8 (13.3)  Other 2 (3.3)  Not applicable 4 (6.7) | cCRT + consolidation immunotherapy 22 (36.7)  Induction chemotherapy + cCRT 20 (33.3)  cCRT + consolidation chemotherapy 9 (15.0)  Induction chemotherapy + cCRT + consolidation chemotherapy 3 (5.0)  sCRT 3 (5.0)  Radical cCRT only 2 (3.3)  sCRT + consolidation immunotherapy 1 (1.7) |
| (12) T1-2, multi-station N2 (non-bulky), hilar + mediastinal nodal stations | CRT 34 (56.7)  Chemotherapy 21 (35.0)  Other 5 (8.3) | cCRT 30 (50.0)  Chemotherapy 14 (23.3)  sCRT 9 (15.0)  Radiotherapy 3 (5.0)  Other 1 (1.7)  Not applicable 3 (5.0) | cCRT + consolidation immunotherapy 23 (38.3)  Induction chemotherapy + cCRT 20 (33.3)  cCRT + consolidation chemotherapy 6 (10.0)  Induction chemotherapy + cCRT + consolidation chemotherapy 2 (3.3)  sCRT 1 (1.7)  Radical cCRT only 5 (8.3)  sCRT + consolidation immunotherapy 2 (3.3)  Other 1 (1.7) |
| (3) T3-4 N2 superior sulcus tumor without chest wall/spinal invasion | CRT 44 (73.3)  Chemotherapy 11 (18.3)  Other 5 (8.3) | Chemotherapy 31 (51.7)  cCRT 15 (25.0)  sCRT 8 (13.3)  Radiotherapy 1 (1.7)  Other 2 (3.3)  Not applicable 3 (5.0) | cCRT + consolidation immunotherapy 23 (38.3)  Induction chemotherapy + cCRT 21 (35.0)  cCRT + consolidation chemotherapy 5 (8.3)  Induction chemotherapy + cCRT + consolidation chemotherapy 3 (5.0)  sCRT 3 (5.0)  Radical cCRT only 5 (8.3) |
| (2) T3-4 N0-1 superior sulcus tumor with chest wall/spinal invasion | CRT 52 (86.7)  Chemotherapy 7 (11.7)  Other 1 (1.7) | Chemotherapy 35 (58.3)  cCRT 16 (26.7)  sCRT 7 (11.7)  Not applicable 2 (3.3) | cCRT + consolidation immunotherapy 20 (33.3)  Induction chemotherapy + cCRT + consolidation chemotherapy 16 (26.7)  cCRT + consolidation chemotherapy 7 (11.7)  Induction chemotherapy + cCRT + consolidation chemotherapy 16 (26.7)  sCRT 3 (5.0)  Radical cCRT only 8 (13.3) |

Percentages are based on non-missing responses.
^a^ Clinical scenarios where less than 80% of respondents selected either Resectable or Unresectable, based on the response to the question: Please assess resectability for this clinical scenario, assuming good performance status (response options: Resectable / Unresectable)
